# Preterm birth, airway microbiome, and the evolutionary origins of asthma

**DOI:** 10.1093/emph/eoag005

**Published:** 2026-04-02

**Authors:** Alisha Khalil, Zaneta Marie Thayer, Luisa Maria Rivera

**Affiliations:** Department of Anthropology, Dartmouth College, 3 Tuck Drive, Hanover, NH 03755, USA; Department of Anthropology, Dartmouth College, 3 Tuck Drive, Hanover, NH 03755, USA; Department of Anthropology, University College London, 14 Taviton Street, WC1H 0BW London, UK

## PRETERM BIRTH AND ASTHMA

Asthma is the most common chronic childhood disease. Preterm birth (<37 weeks), which occurs in ~10% of births globally, increases pediatric asthma risk due to impacts on pulmonary development. Children born very preterm (<31 weeks) face up to a six-fold higher risk of asthma, while those born moderate–late preterm (32–36 weeks) have a 2.5-fold higher risk compared to term infants [1, 2].

## EVOLUTIONARY PERSPECTIVES

Asthma risk following preterm birth potentially reflects two mechanisms: developmental constraint in lung maturation and evolutionary mismatch in airway microbial colonization.

Surfactant, essential for preventing alveolar collapse, reaches optimal levels after 35 weeks, reflecting a conserved evolutionary constraint among amniotes and the fatal nature of preterm birth in ancestral environments [3].

Modern neonatal care enables survival, but disrupts airway colonization by symbiotic microbes (‘Old Friends’). The ‘Old Friends’ hypothesis suggests that loss of symbiotic microbes impairs immunoregulation [4]. In preterm neonates, oxidative stress from supplemental oxygen, mechanical ventilation, and intubation changes airway microbiota, reducing diversity and promoting inflammatory responses (TH1/TH17, elevated IL-1β and TNF-α) [5, 6]. Delayed or reduced breastfeeding among intubated preterm infants further perturbs the airway microbiome. The resulting dysbiosis contributes to bronchopulmonary dysplasia (BPD), a chronic lung disease strongly associated with asthma [6].

## FUTURE IMPLICATIONS

While evolutionary constraints cannot be altered, addressing airway microbial mismatch offers preventative potential for pediatric asthma development. Supporting microbial health through lactation support, such as early administration of mother’s own raw milk (MORM) or frozen donor milk (FDM) inoculated with MORM, may be effective [5, 7]. Probiotic therapies, such as inhaled *Lactobacillus*-based live biotherapeutics, can also help restore microbial balance and reduce neutrophilic inflammation [8]. These strategies extend beyond respiratory disease, as MORM/FDM can also mitigate the risk of neonatal necrotizing enterocolitis and late-onset sepsis [7]. Together, these interventions may mitigate dysbiosis and immune dysregulation in preterm neonates, lowering asthma risk and the associated global health burden.
